# Cell-specific regulation of insulin action and hepatic fibrosis by CEACAM1

**DOI:** 10.20517/mtod.2024.48

**Published:** 2024-09-26

**Authors:** Basel G. Aldroubi, John A. Najjar, Tya S. Youssef, Carl E. Rizk, Basil A.M. Abuamreh, Karl Aramouni, Hilda E. Ghadieh, Sonia M. Najjar

**Affiliations:** 1 Department of Biomedical Sciences, Heritage College of Osteopathic Medicine, Ohio University, Athens, OH 45701, USA.; 2 Department of Pathology, College of Medicine and Life Sciences, University of Toledo, Toledo, OH 43614, USA.; 3 Department of Biomedical Sciences, Faculty of Medicine and Medical Sciences, University of Balamand, Al-Koura PO box 100 Tripoli, Kalhat, Lebanon.; 4 Department of Medicine, Faculty of Medicine, American University of Beirut, Beirut 1107-2020, Lebanon.; 5 Diabetes Institute, Heritage College of Osteopathic Medicine, Ohio University, Athens, OH 43614, USA.

**Keywords:** Insulin action, insulin resistance, insulin clearance, hepatic steatosis, hepatic fibrosis, liver injury

## Abstract

The incidence of metabolic dysfunction-associated steatotic liver disease (MASLD) has reached an epidemic rise worldwide. The disease is a constellation of a broad range of metabolic and histopathologic abnormalities. It begins with hepatic steatosis and progresses to metabolic dysfunction-associated steatohepatitis (MASH), including hepatic fibrosis, apoptosis, and cell injury. Despite ample research effort, the pathogenesis of the disease has not been fully delineated. Whereas insulin resistance is implicated in the early stages of the disease, its role in hepatic fibrosis remains controversial. We have focused our studies on the role of carcinoembryonic antigen-related cell adhesion molecule 1 (CEACAM1) in hepatocytes and endothelial cells in the metabolic and histopathological dysregulation in MASH. Patients with MASH exhibit lower hepatic CEACAM1 with a progressive decline in hepatocytes and endothelial cells as the fibrosis stage advances. In mice, conditional deletion of CEACAM1 in hepatocytes impairs insulin clearance to cause hyperinsulinemia-driven insulin resistance with steatohepatitis and hepatic fibrosis even when mice are fed a regular chow diet. In contrast, its conditional deletion in endothelial cells causes inflammation-driven hepatic fibrosis without adversely affecting metabolism (mice remain insulin-sensitive and do not develop hepatic steatosis). Thus, this review provides *in vivo* evidence that supports or discards the role of insulin resistance in liver injury and hepatic fibrosis.

## Graphical Abstract

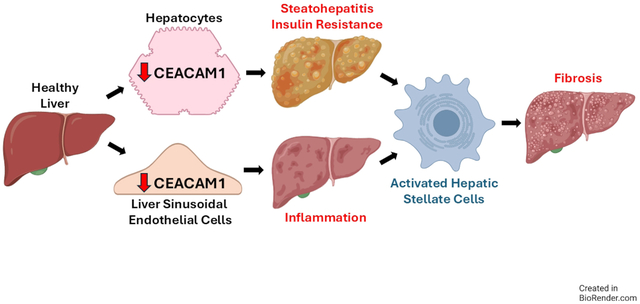

## INTRODUCTION

Metabolic dysfunction-associated steatotic liver disease (MASLD), previously known as non-alcoholic fatty liver disease (NAFLD)^[1]^, is a heterogeneous disease^[2]^. Its broad spectrum spans simple steatosis to steatohepatitis to hepatic fibrosis, which marks its progression to metabolic dysfunction-associated steatohepatitis (MASH), together with apoptosis and cell injury. If uncontrolled, liver fibrosis can progress to cirrhosis and hepatocellular carcinoma^[3]^. In fact, this disease has topped the list of abnormalities that lead to liver transplant in the US and its incidence has grown in children and adolescents, especially of Mexican descent^[4–7]^.

MASLD/MASH is spreading worldwide in parallel to the increasing incidence of metabolic syndrome (MetS), a constellation of diseases that include visceral obesity, hyperinsulinemia/type 2 diabetes, hypertension, and atherosclerosis^[8]^. The reasonably high degree of overlap in its mechanistic underpinning with atherosclerosis has led to their identification as twin diseases^[9,10]^.

Liver fibrosis evolves when hepatic stellate cells (HSCs) are activated to produce collagen fibers that escape fibrolysis^[11]^. Its rapid spread stems in part from our limited understanding of its pathogenesis, which has slowed down progress in developing Food and Drug Administration (FDA)-approved targeted therapies. Lifestyle changes (diet, exercise and restricted alcohol intake) remain the cornerstone of treatment of this disease, at least at its early stages^[2]^. More recently, progress has been made and several metabolism-improving drugs have been developed and are now at stage 2a/3 clinical trials. These include thyroid hormone receptor beta-selective agonists and fatty acid synthase (FASN) inhibitors^[12]^. With insulin resistance playing a major role in the pathogenesis of MetS^[13,14]^, repurposing insulin sensitizers and incretins glucagon-like-peptide-1 receptor (GLP-1R)-agonists has become an accepted therapeutic strategy, particularly at the early stages of the disease^[15]^. However, their use in curbing hepatic fibrosis remains controversial. Whereas GLP-1R agonists (exenatide, liraglutide, semaglutide) improve glucose disposal in response to increased insulin secretion, reduce food intake and body weight, and ameliorate insulin resistance and hepatic steatosis^[16]^, their efficacy in late stages of metabolic liver injury remains questionable^[17]^. To date, phase 2 clinical trials have excluded an antifibrotic effect for liraglutide^[18]^ and semaglutide^[19]^. The limited efficacy of these drugs in the resolution of hepatic fibrosis is consistent with the fact that not all patients with advanced hepatic fibrosis are insulin-resistant^[20–24]^. In fact, hepatic fibrosis in some patients likely stems from genetic predisposition, inflammatory diseases, and others.

If MASLD/MASH is a prominent feature of MetS, with insulin resistance being at its foundation, it is logical to assign a significant role for insulin resistance in its pathogenesis. Because MASLD/MASH is the liver manifestation of MetS, we will focus this review on hepatic insulin resistance. In the liver, hepatic insulin clearance plays a critical role in regulating insulin and lipid metabolism and subsequently, insulin action. This is bolstered by the emergence of reduced insulin clearance as a risk factor for MetS, especially among African Americans, Native Americans, and Hispanics^[25,26]^.

## INSULIN ACTION

Insulin is secreted from pancreatic β-cells in response to stimuli and exerts its effects on several target tissues, including classically known targets: the liver where it suppresses endogenous glucose production, skeletal muscle where it promotes glucose uptake, and adipose tissue where it mainly promotes fat storage and contributes to glucose uptake.

Insulin action is mediated by insulin binding to the α-subunit of its receptor (IRα) followed by trans-activation of the tyrosine kinase of its β-subunit (IRβ). This initiates a cascade of phosphorylation/dephosphorylation that leads to a myriad of insulin actions in a cell- and tissue-specific manner^[27–30]^. Whereas insulin signaling mediates insulin action, this process is regulated by circulating insulin level, which is determined by the net balance of insulin secretion from pancreatic β-cells and its clearance from the circulation, mainly in liver and to a lower extent in kidney^[31,32]^.

## INSULIN CLEARANCE

We and others have presented several reviews on insulin clearance and its role in regulating insulin action^[31,33–35]^. In brief, receptor-mediated insulin uptake and degradation constitute the main mechanism of insulin extraction. Endogenous insulin is cleared mostly in hepatocytes and, to a lower extent, in renal proximal tubule cells. In response to stimuli, insulin is secreted in pulses from pancreatic β-cells into the portal vein to passively and rapidly reach hepatocytes via fenestrae in the capillaries in the liver sinusoid. This leads to the binding of insulin to its receptor and its delivery to its degradation processes. Up to 70%−80% of insulin is cleared during its first pass through hepatocytes. In this manner, hepatic insulin clearance regulates the amount of insulin reaching its peripheral targets, such as skeletal muscle and white adipose tissue, where its delivery is tightly controlled by endothelial cells lining systemic vessels^[33,36,37]^.

**Figure 1.**
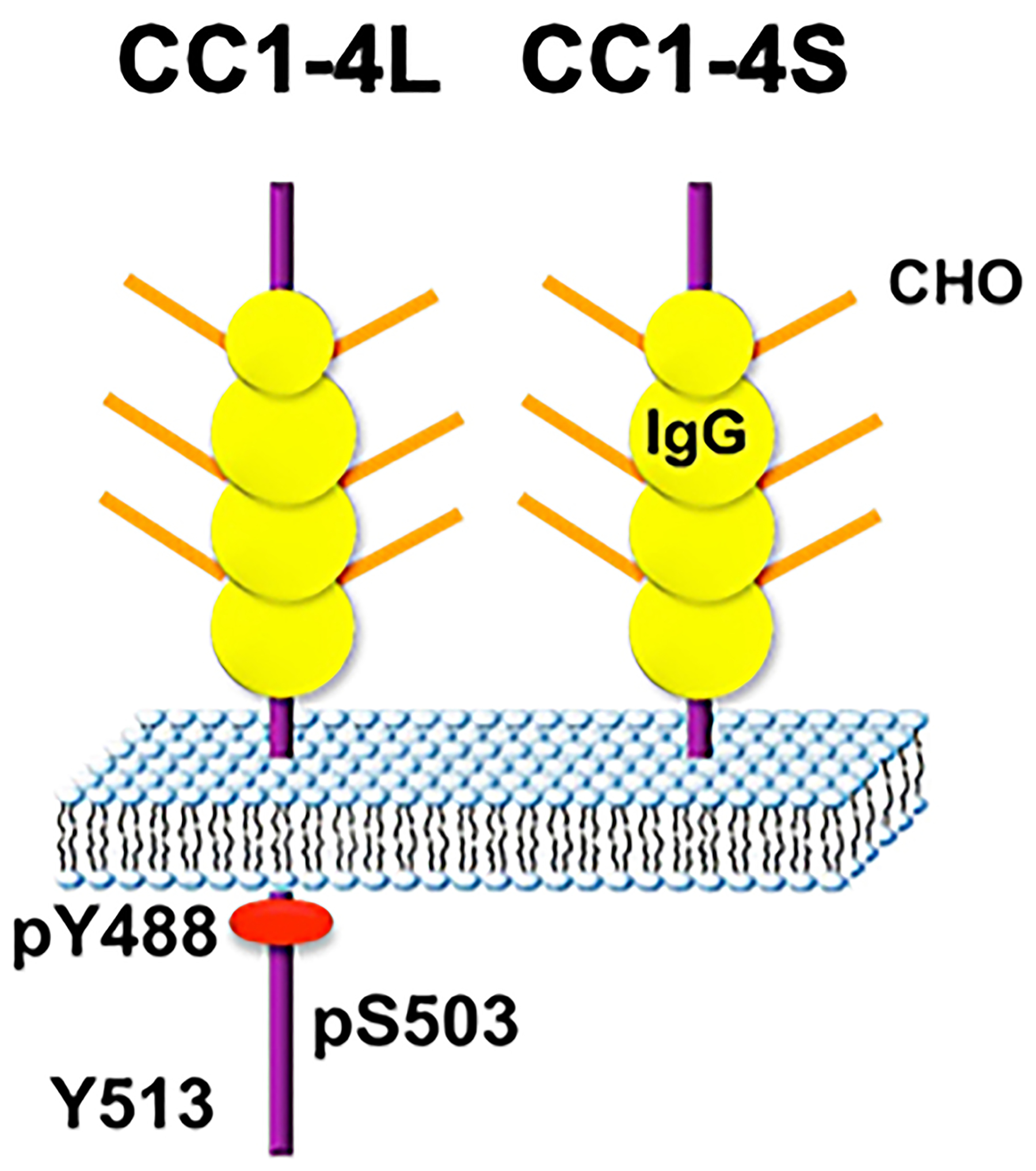
Alternative spliced variants of CEACAM1. In the liver, CEACAM1 is expressed as 2 spliced isoforms resulting from deletion or inclusion of Exon 7. The long isoform, denoted CC1-4L, contains a longer intracellular tail than the short (CC1-4S) (71 *vs*. 11 a.a.). Its cytoplasmic tail includes serine and tyrosine phosphorylation sites. Serine503 residue must be intact for Tyrosine488 to undergo phosphorylation by the insulin receptor tyrosine kinase. Both forms have 4 IgG loops and several CHO on their extracellular domains. CEACAM1: Carcinoembryonic antigen-related cell adhesion molecule 1; CEACAM1-4L: CC1-4L; CHO: carbohydrate chains.

Insulin binding to its receptor causes its dimerization and trans-activation of its tyrosine kinase in the intracellular domain to phosphorylate, among other substrates, the carcinoembryonic antigen-related cell adhesion molecule 1 (CEACAM1). CEACAM1 is a ubiquitous plasma membrane glycoprotein with a predominant expression in the liver, but not in skeletal muscle or adipose tissue, among classical insulin target tissues. In hepatocytes, it is expressed as two alternatively spliced isoforms differing by the presence or absence of 60/71 amino acids (based on the rat sequence) and 60/73 amino acids (based on the mouse sequence) of its well-conserved intracellular tail that harbors its phosphorylation sites^[38,39]^ [Figure 1]. The long isoform (CEACAM1-4L) is expressed on both the sinusoidal and the bile canalicular domains of hepatocytes, whereas the short isoform (CEACAM1-4S) that lacks Serine503 and Tyrosine513 in the rat (or 515 in the mouse) is expressed exclusively in the bile canalicular domain^[40]^.

**Figure 2.**
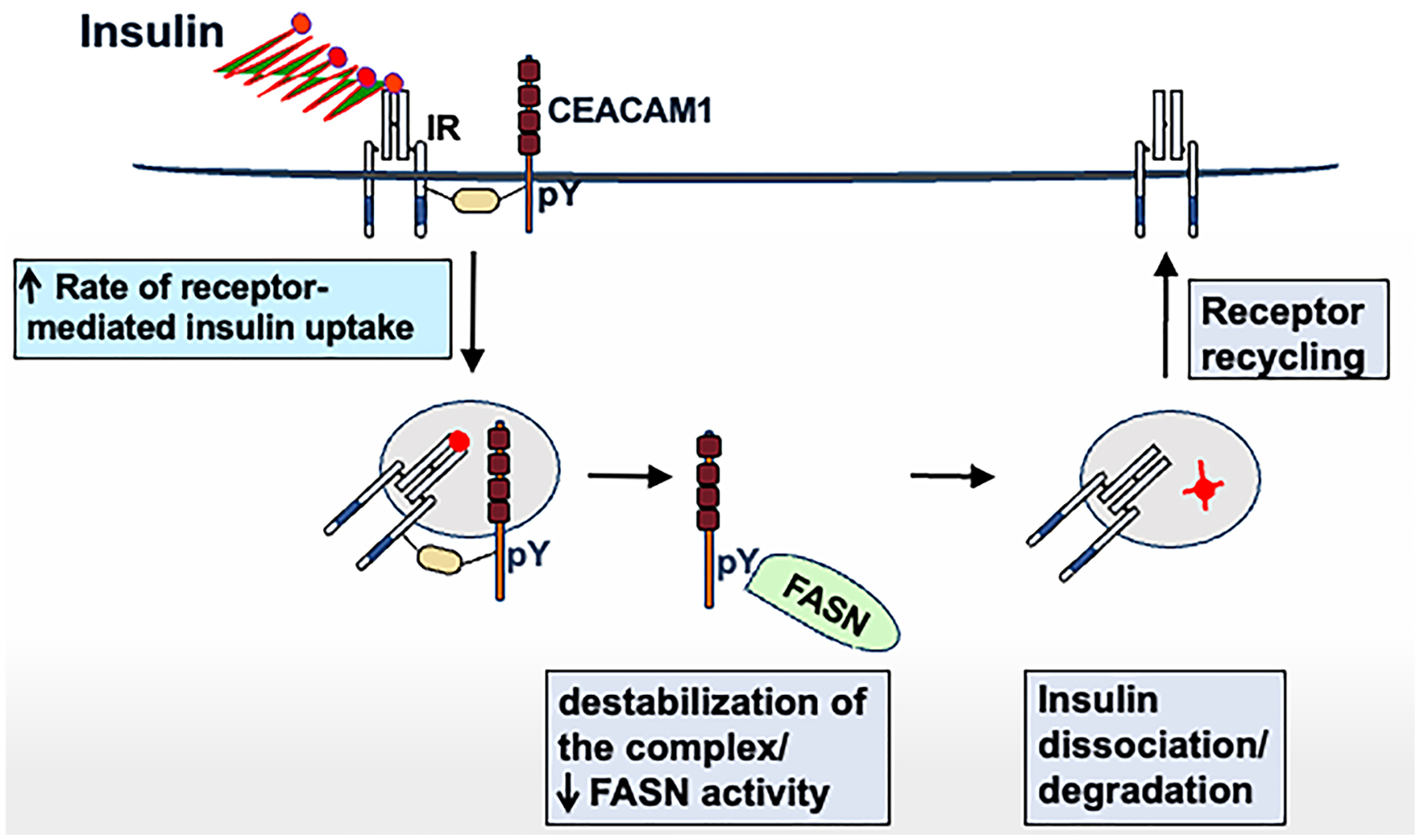
The double role of CEACAM1 in hepatocytes. In response to stimuli, insulin is released from pancreatic β-cells in pulses. This would stimulate CEACAM1 phosphorylation by the activated IR in hepatocytes. Phosphorylated CEACAM1 stabilizes the IR-insulin endocytosis complex and induces the rate of its uptake and insulin delivery to lysosomal degradation. By binding to FASN, expressed at high levels in the perinuclear region, CEACAM1 dissociates from the complex to allow the detachment of insulin from its receptor in the acidic environment of endosomes to undergo degradation. This also leads to suppression of FASN activity. In this manner, FASN activity in hepatocytes is kept at minimal under normal physiologic conditions despite its high levels resulting from increased transcription by the physiologic high insulin in the portal circulation. Thus, the pulsatility of secreted insulin and ensuing CEACAM1 phosphorylation by activated insulin receptors protect the liver against the otherwise lipogenic effect of physiologic high insulin in the portal vein. The upward arrow ↑ indicates an increase, and the downward arrow ↓ indicates a decrease. CEACAM1: Carcinoembryonic antigen-related cell adhesion molecule 1; IR: insulin receptor; FASN: fatty acid synthase.

Upon its phosphorylation by IRβ in response to acute insulin pulses, CEACAM1-4L (hereafter referred to as CEACAM1) takes part in the insulin-IR complex to stabilize it and increase the rate of its cellular uptake and insulin’s delivery to the degradation process^[33,41]^ [Figure 2]. For insulin to undergo degradation in the acidic environment of late endosomes, it must dissociate from its receptor. This is achieved by the dissociation of CEACAM1 from the endocytosis complex following its reciprocal binding to FASN that is highly expressed in the perinuclear region. The receptor undergoes recycling, whereas the CEACAM1/FASN association mediates the repression of FASN activity in response to insulin^[42,43]^. In this manner, we demonstrated a novel acute negative effect of insulin on *de novo* lipogenesis (DNL) linked to promoting insulin clearance in hepatocytes in response to acute rises of insulin in the portal vein. In other words, CEACAM1 phosphorylation by IRβ bestows protection against the otherwise lipogenic effect of the physiologic high insulin level in the portal circulation^[27]^ and this is achieved by promoting insulin clearance and suppressing DNL. This negative acute physiologic effect of insulin on DNL is in contrast to its permissive chronic effect when supraphysiologic levels of insulin activate SREBP-1c to induce the transcription of lipogenic genes, such as FASN^[44]^ and, subsequently, promote DNL. Together with diminished pulsatility of insulin and downregulated insulin receptor number, chronic hyperinsulinemia increases DNL and causes hepatic insulin resistance. Thus, we posited that hepatic insulin resistance is inclusive of the inability of insulin to acutely suppress lipogenesis as well as gluconeogenesis. Thus, we propose that hepatic insulin resistance is not as selective for gluconeogenesis as commonly believed. This is supported by a recent report showing that in patients with MASLD, hepatic insulin resistance is not pathway-selective^[45]^.

## REDUCED HEPATIC INSULIN CLEARANCE, INCREASED LIPOGENESIS AND HEPATIC INSULIN RESISTANCE: CEACAM1 IS A MOLECULAR LINK

**Figure 3.**
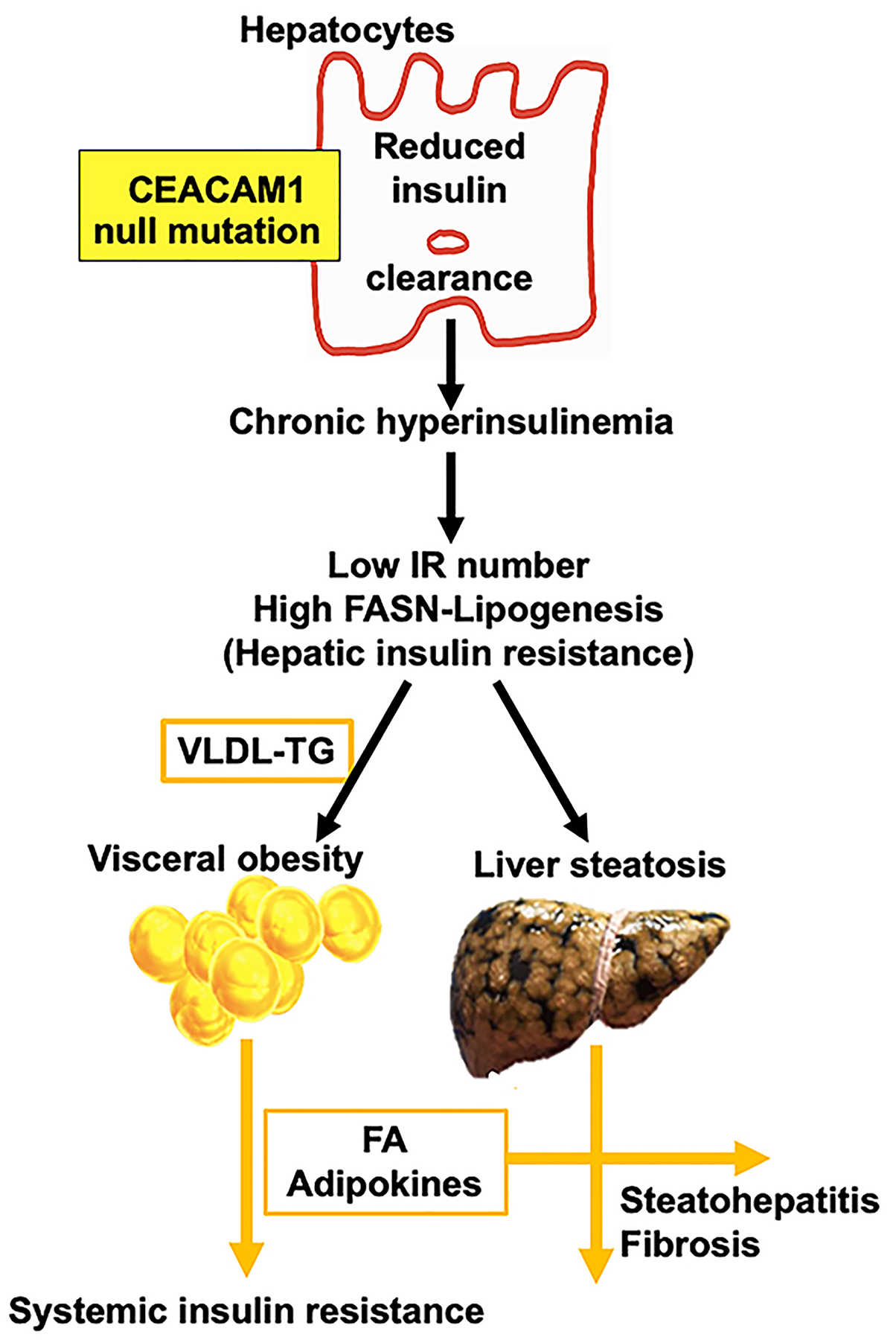
Loss of CEACAM1 in hepatocytes causes insulin resistance and hepatic fibrosis. Loss of CEACAM1 in hepatocytes impairs insulin clearance, which causes hyperinsulinemia-driven hepatic insulin resistance and *de novo* lipogenesis (steatosis). Redistribution of VLDL-triglycerides to white adipose tissue causes visceral obesity and, eventually, excessive release of FA and adipokines, both of which could lead to systemic insulin resistance. In addition to fat accumulation in the liver, adipokines can alter the inflammatory milieu of the liver and steatohepatitis emerges. Both FA and IL-6 could transactivate EGFR in HSCs to mediate their activation and cause collagen production and hepatic fibrosis [Cf Figure 4]. CEACAM1: Carcinoembryonic antigen-related cell adhesion molecule 1; FA: fatty acids; EGFR: epidermal growth factor receptor; HSCs: hepatic stellate cells; IR: insulin receptor; FASN: fatty acid synthase.

Insulin resistance, manifested by chronic hyperinsulinemia, is the hallmark of MetS. It occurs when insulin response in target tissues is compromised. It has been established that chronic hyperinsulinemia in insulin resistance implicates both β-cell dysfunction and impaired insulin clearance^[46]^. However, the cause-effect relationship remains a subject of debate. It is commonly believed that primary insulin resistance, when associated with visceral obesity, is compensated for by increased insulin secretion and reduced insulin clearance; the latter serves to limit compensatory insulin release and prolong the life/function of the β-cell^[47]^. Alternatively, it is also possible that reduced hepatic insulin clearance causes chronic hyperinsulinemia, which, in turn, leads to downregulation of the insulin receptor, and subsequently hepatic insulin resistance^[48–50]^. Hyperinsulinemia also induces DNL followed by the assembly of very-low-density lipoprotein (VLDL)-triglycerides and redistribution to white adipose tissue for fat storage. Eventually, the release of adipokines and excessive lipolysis-derived free fatty acids (FA) together cause systemic insulin resistance [Figure 3]. Accordingly, it is conceptually possible that reduced hepatic insulin clearance could cause hyperinsulinemia-driven hepatic insulin resistance and fat accumulation in the liver (hepatic steatosis). This paradigm is in line with the emergence of reduced insulin clearance as a risk factor for metabolic dysregulation^[51–53]^.

As detailed below, this alternative paradigm has been bolstered by the phenotype of mice with liver-specific loss-of-function of *Ceacam1*, the gene encoding CEACAM1 (L-SACC1 and *AlbCre+Cc1*^*fl/fl*^ mutants)^[54,55]^, and by the reversal of systemic insulin resistance and hepatic steatohepatitis in global *Ceacam1* null mice when CEACAM1 is exclusively reconstituted in hepatocytes^[56]^. This highlights a key role for CEACAM1-dependent insulin clearance pathways in maintaining insulin sensitivity and limiting hepatic steatosis by promoting hepatic insulin clearance and mediating the anti-lipogenic effect of acutely released insulin in hepatocytes.

According to this paradigm, reduced hepatic insulin clearance causes secondary hepatic insulin resistance and steatosis. However, delineating this paradigm in patients has been challenged by at least two main factors: 1) both insulin action and clearance depend on the rapid insulin binding to its receptor, and 2) in the Western world, most patients exhibit systemic insulin resistance with prominent abdominal obesity that could prohibit a fair assessment of an earlier onset of reduced insulin clearance. Accordingly, studies in Japanese subjects who exhibit milder abdominal obesity showed that impaired insulin clearance causes hepatic insulin resistance and steatosis^[57]^. Moreover, Bril *et al*. from the University of Florida reported that hyperinsulinemia in patients with MASLD is caused primarily by reduced insulin clearance rather than increased insulin secretion^[58]^. Thus, clinical research employing a more sensitive assessment of insulin clearance is needed to determine in human subjects that reduced insulin clearance can cause insulin resistance and is not just a consequence thereof.

## IMPAIRED INSULIN CLEARANCE PRECEDES INFLAMMATION IN THE PATHOGENESIS OF DIET-INDUCED INSULIN RESISTANCE

High-fat feeding causes a progressive decline in hepatic CEACAM1 expression by a peroxisome proliferator-activated receptor alpha (PPARα)-dependent mechanism^[59]^. This provides a positive feedback mechanism on fatty acid β-oxidation (FAO) while maintaining intact insulin clearance and insulin sensitivity within the first 2 weeks of diet initiation in parallel to ≤ 50% loss of *Ceacam1* expression^[59]^. The mild decrease in hepatocytic CEACAM1 expression relieves FASN from the negative effect of CEACAM1 to lower malonyl-CoA level and its inhibition of carnitine palmitoyltransferase I (CPT1) activity and promote mitochondrial transport of long chain fatty acyl-CoA for FAO. Sustained high fat intake for > 3 weeks causes a loss of CEACAM1 by > 60%, at which point, insulin clearance is impaired and hepatic insulin resistance develops preceding inflammation^[60]^. At this point, re-esterification dominates over FAO and DNL contributes to hepatic steatosis. These observations were bolstered by the reversal of diet-induced insulin resistance and hepatic steatosis by acute adenoviral-mediated delivery of wild-type CEACAM1 to the liver^[61]^ or by forced liver-specific over-expression of rat transgene using human Apolipoprotein A1 promoter that is induced by high-fat feeding^[60]^.

## LOSS OF CEACAM1 IN THE LIVER OF PATIENTS WITH MASH

Hepatic CEACAM1 levels were significantly reduced in 29% of South Korean obese subjects with insulin resistance and hepatic steatosis independently of diabetes^[62]^. Likewise, in collaboration with Drs. A. Zarrinpar and S. Duarte from the University of Florida, we have shown that hepatic CEACAM1 levels are lower in liver biopsies of patients with MASH than normal subjects regardless of gender, ethnicity and race^[63]^. Furthermore, hepatic CEACAM1 levels progressively decline with the advancement of hepatic fibrosis stage in patients with MASLD/MASH^[63]^.

CEACAM1 is expressed in all liver cells, which are all virtually involved in hepatic stellate cell activation to cause fibrosis. Thus, we have begun by deleting the *Ceacam1* gene individually in murine hepatocytes and endothelial cells (highest and second highest site of CEACAM1 expression, respectively) to evaluate its cell-specific role in the pathogenesis of hepatic fibrosis and delineate the underlying mechanisms. We summarize these findings below.

## LOSS OF CEACAM1 IN HEPATOCYTES LINKS HEPATIC INSULIN RESISTANCE AND STEATOSIS TO FIBROSIS AND LIVER INJURY

Recapitulating the human disease, mice with global deletion of *Ceacam1* (*Cc1*−*/*−) exhibited hyperinsulinemia-driven insulin resistance, steatohepatitis and hepatic fibrosis even when fed a regular chow diet^[60]^. Fed a high-fat diet, hepatic fibrosis was amplified, and mice developed liver injury and apoptosis^[64–67]^.

Deleting CEACAM1 exclusively in hepatocytes (as in *AlbCre+Cc1*^*fl/fl*^ mice) impaired hepatic insulin clearance at 2–3 months of age, followed by hyperinsulinemia-driven hepatic insulin resistance and steatosis at ~6 months of age. Pair-feeding experiments showed that the increase in food intake of *AlbCre+Cc1*^*fl/fl*^ mice at 7 months of age (partly driven by elevated hyperinsulinemia-driven induction of hypothalamic FASN level and activity) contributed to their visceral obesity and excessive release of free FA and adipokines, ultimately causing systemic insulin resistance starting at 8–9 months of age^[54]^.

Histological analysis showed that in addition to increased accumulation of fat droplets, there was also an increase in inflammatory infiltration in the parenchyma of liver sections of 8-month-old *AlbCre+Cc1*^*fl/fl*^ mice. In addition to steatohepatitis and hepatic injury, Sirius Red staining indicated hepatic fibrosis in these mice, even when they were fed a regular chow diet^[63]^. High fat intake caused apoptosis and amplified liver injury^[63]^. These observations were corroborated by the reversal of the metabolic dysfunction and hepatic fibrosis together with other features of MASLD/MASH in global *Cc1*^−/−^ nulls by liver-specific reconstitution of *Ceacam1* even when mice were fed a high-fat diet^[56,66]^.

Hepatic fibrosis is characterized by initial deposition of perisinusoidal collagen, followed by portal and bridging fibrosis^[68]^. This results from the activation of HSCs situated in the Space of Disse between hepatocytes and liver sinusoidal endothelial cells (LSECs). When quiescent, HSCs store vitamin A^[69]^ in response to activated peroxisome proliferator-activated receptor gamma (PPARγ). Following their transdifferentiation into proliferative, inflammatory myofibroblasts with enhanced extracellular matrix production, HSCs lose their PPARγ and retinoids^[70]^, while they reciprocally gain PPARβ/δ^[71,72]^ that undergo activation by the released FA (all-trans retinoic acid^[73]^ and polyunsaturated FA^[74]^).

**Figure 4.**
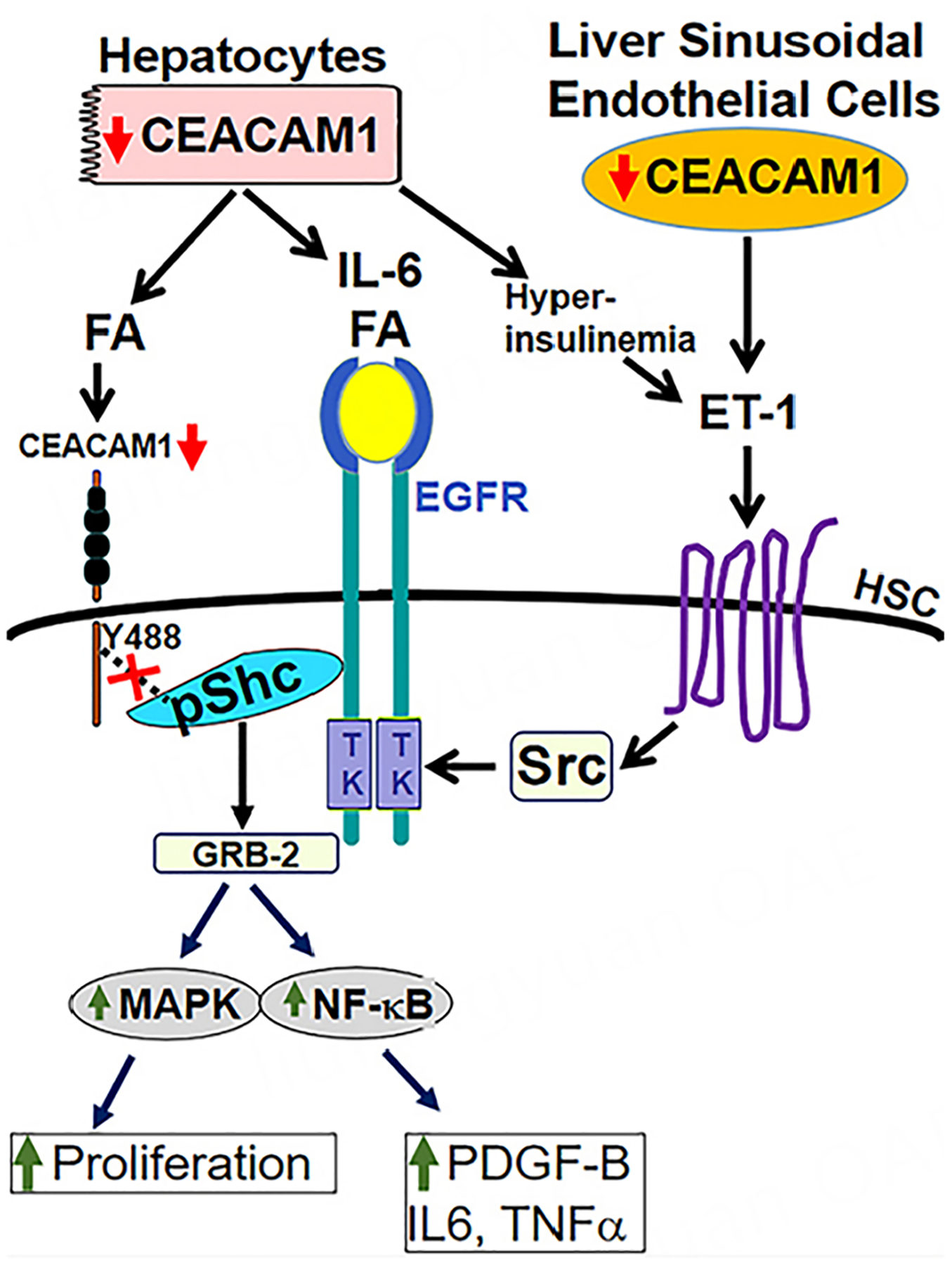
Loss of CEACAM1 in hepatocytes and endothelial cells activates HSCs. As in the legend of Figure 3, the loss of CEACAM1 in hepatocytes causes the release of FA, IL-6 and ET-1 that could activate EGFR in HSCs. Moreover, FA activate PPARβ/δ to reduce the transcription of CEACAM1, which, upon its phosphorylation by EGFR, sequesters Shc to counter cell proliferation. Reduced levels of CEACAM1 in HSCs would consequently elevate the coupling of Shc to the activated EGFR and amplification of Shc/MAPK and Shc/NF-kB downstream signaling. The latter leads to the transcriptional activation of several cytokines and to PDGF-B pro-fibrogenic factor. Loss of CEACAM1 in endothelial cells leads to increased production of ET-1, a vasoconstrictor that applies its pro-fibrogenic effects by binding to its A receptor on the surface membrane of HSCs to transactivate EGFR via Src kinase. Thus, the loss of CEACAM1 in hepatocytes and endothelial cells merges at the level of activation of EGFR to cause myofibroblastic transformation of HSCs and hepatic fibrosis. The upward green arrow ↑ indicates an increase, and the downward red arrow ↓ indicates a decrease. CEACAM1: Carcinoembryonic antigen-related cell adhesion molecule 1; HSCs: hepatic stellate cells; FA: fatty acids; IL-6: interleukin-6; ET-1: endothelin-1; EGFR: epidermal growth factor receptor; PDGF-B: platelet-derived growth factor subunit B.

Among other mechanisms, we have recently shown that these FA activate the epidermal growth factor receptor (EGFR) on the surface membrane of HSCs to cause their myofibroblastic transformation^[63,75,76]^ [Figure 4]. In addition to FA^[75]^, IL-6 also transactivates EGFR^[77]^. Consistently, deleting CEACAM1 from hepatocytes caused steatosis and visceral obesity, both of which could partially alter the inflammatory milieu of the liver through the release of IL-6 among other interleukins^[78]^. Visceral obesity leads to lipolysis-derived FA that could activate EGFR in liver cells following their passage through the portal vein^[79]^. Moreover, hyperinsulinemia caused by deleting CEACAM1 in hepatocytes induced the production of Endothelin-1, which transactivates EGFR via Src kinase, as we have shown^[63]^. Consistently, plasma Endothelin-1 levels were elevated in *AlbCre+Cc1*^*fl/fl*^ mice starting at 6 months of age preceding hepatic fibrosis. Additionally, media transfer experiments demonstrated that Endothelin-1 release from mutant hepatocytes played a significant role in activating wild-type HSCs^[63]^. As summarized in Figure 4, both IL-6 and FA activate EGFR in HSCs. With FA suppressing *Ceacam1* expression in HSCs by activating PPARβ/δ, as we have recently shown^[75]^, sequestration of Shc by CEACAM1 upon its phosphorylation by EGFR is diminished, leading to its amplified coupling to EGFR. This activates Shc/MAPK proliferation and Shc/NF-kB inflammatory pathways. The latter leads to increased autocrine expression of several transcriptional targets of NF-kB such as TNFα and IL-6 adipokines^[63]^ and of PDGF-B and its receptor. Together, these contribute to the activation of HSCs and their myofibroblastic transformation. This was demonstrated by the activation of wild-type HSCs by conditioned media from hepatocytes isolated from *AlbCre+Cc1*^*fl/fl*^ mice independently of other cells and without injuring endothelial cells^[63]^.

Similarly, L-SACC1 mice with liver-specific inactivation of CEACAM1 (overexpressing a dominant-negative phosphorylation-defective S503A isoform that evades sinusoidal localization)^[40]^ displayed visceral obesity, insulin resistance, steatohepatitis and spontaneous hepatic fibrosis^[55]^ in addition to exaggerated hepatic fibrosis when fed a high-fat diet^[80]^. On the other hand, overexpressing CEACAM1 in hepatocytes protected mice against the metabolic and liver histological abnormalities caused by high-fat feeding for 4 months^[60]^. It also protected mice from developing fibrosis in adipose tissue in response to long-term high fat intake^[81]^.

Together, this demonstrates that CEACAM1 in hepatocytes plays a key role in maintaining insulin sensitivity by promoting insulin clearance. It also prevents hepatic steatohepatitis as well as fibrosis and liver injury. Mice with hepatocytes-specific deletion (*AlbCre*+*Cc1*^*fl/fl*^) or inactivation (L-SACC1) of *Ceacam1* provide an *in vivo* demonstration that hepatic inflammation and fibrosis can be associated with visceral obesity, insulin resistance, and hepatic steatosis. This supports a potentially significant role of reduced insulin clearance as a risk factor for hepatic fibrosis associated with MetS.

## LOSS OF CEACAM1 IN ENDOTHELIAL CELLS CAUSES ENDOTHELIN-1–DRIVEN HEPATIC FIBROSIS IN THE ABSENCE OF HEPATIC INSULIN RESISTANCE OR STEATOSIS

In contrast to *AlbCre*+*Cc1*^*fl/fl*^, *VECadCre*+*Cc1*^*fl/fl*^ mice with exclusive loss of *Ceacam1* in endothelial cells did not display impairment of insulin clearance or insulin resistance^[82]^. Consistent with normo-insulinemia, *VECadCre*+*Cc1*^*fl/fl*^ mice did not develop hepatic steatosis^[82]^. However, like hepatocytes, loss of endothelial CEACAM1 restricted Shc sequestration to increase its reciprocal coupling to VEGFR and activation of downstream NF-kB pathways. This led to hepatic and systemic inflammation in *VECadCre*+*Cc1*^*fl/fl*^ mice, as manifested by a remarkable rise in plasma IL-6 and TNFα levels at 8 months of age^[76]^. Activation of NF-kB also led to increased transcription of Endothelin-1 and its autocrine production, followed by a spike in its circulating levels. In light of the pro-fibrogenic role of Endothelin-1, *VECadCre*+*Cc1*^*fl/fl*^ mice developed hepatic fibrosis with bridging chicken-wire deposition of collagen fibers in their liver parenchyma at 8 months of age even when fed a regular chow diet^[76]^. The role of Endothelin-1 in this phenotype was bolstered by the reversal of fibrosis in *VECadCre*+*Cc1*^*fl/fl*^ mice with combined endothelial loss of *Ceacam1(Cc1*) and *Endothelin-1(Et1)* genes. Moreover, conditioned media from *VECadCre*+*Cc1*^*fl/fl*^, but not *VECadCre*+*Et1.Cc1*^*fl/fl*^ primary liver endothelial cells, activated wild-type HSCs^[76]^. In keeping with the predominant expression of CEACAM1 in LSECs relative to the general endothelial pool in the liver, LSECs of *VECadCre*+*Cc1*^*fl/fl*^ single, but not *VECadCre*+*Et1.Cc1*^*fl/fl*^ double mutants, manifested cell injury, characteristic of hepatic fibrosis^[76]^. This was accompanied by increased Endothelin-1 production from LSECs. It is likely that Endothelin-1 exerted its pro-fibrogenic effect by transactivating EGFR in HSCs derived from *VECadCre*+*Cc1*^*fl/fl*^ single, but not *VECadCre*+*Et1.Cc1*^*fl/fl*^ double mutants^[76]^. Together, these data demonstrated that endothelial CEACAM1 plays a key role in preventing hepatic fibrogenesis by reducing autocrine Endothelin-1 production. Thus, *VECadCre*+*Cc1*^*fl/fl*^ mice provided an *in vivo* demonstration that hepatic fibrosis can result from inflammation in the absence of insulin resistance and hepatic steatosis.

Recapitulating the phenotype in mice, immunohistochemical analyses of liver tissue biopsies from patients with MASH diagnosis receiving liver transplant revealed lower endothelial CEACAM1 levels than adult patients undergoing bariatric surgery^[76]^. Moreover, endothelial CEACAM1 expression gradually declined with the advanced hepatic fibrosis stage and in parallel to the progressive increase in plasma Endothelin-1 levels of patients with MASH^[76]^. Furthermore, single-cell sequencing analysis of liver cells showed lower CEACAM1 and, reciprocally, higher Endothelin-1 mRNA levels in LSECs of patients with advanced fibrosis as compared to normal subjects^[76]^.

## LOSS OF CEACAM1 RATHER THAN INSULIN RESISTANCE PLAYS A KEY ROLE IN THE PATHOGENESIS OF HEPATIC FIBROSIS

Our data demonstrated that whereas the loss of CEACAM1 in hepatocytes causes hyperinsulinemia-driven hepatic insulin resistance and steatosis in addition to hepatic inflammation and fibrosis, its endothelial loss causes inflammation-driven hepatic fibrosis in the absence of insulin resistance and hepatic steatosis. This demonstrates that insulin resistance is likely implicated in the early stages of MASLD/MASH, and that the loss of CEACAM1 in the two most prominent cell populations in the liver constitutes a unifying mechanism underlying hepatic fibrosis (and inflammation) in the advanced stages of the disease. In support of this notion, insulin sensitizers, like PPARγ agonists, and incretins, like GLP-1 receptor agonists, individually or combined, have been used for the treatment of MASH, at least in the early stages of the disease. Both classes of drugs induced the transcription of *Ceacam1* by directly binding to the well-conserved PPRE/RXR sequence on its promoter, as shown by a chromatin immunoprecipitation assay in human hepatoma HepG2 cells treated with rosiglitazone, a PPARγ agonist, or exenatide, a GLP-1R agonist^[83]^. Consistently, exenatide treatment for 6 h induced GLP-1R mRNA levels in HepG2 cells, followed by inducing CEACAM1 and PPARγ mRNA levels after 12 h of treatment. This positive effect of exenatide was mediated by its receptor, as demonstrated by the prevention of these effects by pre-incubating cells with exendin 9–39 GLP-1R antagonist^[83]^. Moreover, treating mice with Exenatide reversed insulin resistance together with hepatic steatosis and fibrosis in wild-type but not in *AlbCre+Cc1*^*fl/fl*^ mice fed a high-fat diet. Thus, a CEACAM1-targeted therapeutic approach could constitute an effective strategy against hepatic fibrosis while it ameliorates insulin resistance in patients with this metabolic abnormality.

## STRENGTHS AND LIMITATIONS

The data above demonstrate that the loss of CEACAM1 in hepatocytes and endothelial cells constitutes a unifying mechanism underlying hepatic fibrosis in mice independently of metabolic regulation. However, more studies are needed to delineate the independent role of CEACAM1 in other liver cells, such as Kupffer cells, before we could formulate a CEACAM1-based unifying mechanism against hepatic fibrosis. It is worth mentioning that CEACAM1 is at the crossroads of the regulation of metabolic and immune response in liver injury, as we have previously reviewed^[84]^. Moreover, in both mice and humans, hepatic CEACAM1 expression correlated negatively with activation of innate and adaptive immune responses, demonstrating that CEACAM1 expression indicates donor liver quality and prevents early orthotopic transplantation injury^[85]^.

Because CEACAM1 is gradually lost in hepatocytes and endothelial cells in MASH patients as fibrosis progresses, the data promote CEACAM1 induction as a potential therapeutic strategy. Considering the potential confounding metabolic effects of PPARγ and GLP-1R agonists, it would be of utmost importance to identify effectors that specifically induce *Ceacam1* promoter activity in an attempt to develop CEACAM1-targeted therapy with high efficacy against hepatic fibrosis and limited off-target effects.

## CONCLUSION

In summary, loss of CEACAM1 in the two most dominant cells in the liver causes hepatic fibrosis with inflammation. In contrast to endothelial cells, its loss in hepatocytes also causes hepatic insulin resistance and steatosis, consistent with its role in promoting insulin clearance in these cells. Thus, loss of CEACAM1 provides an *in vivo* demonstration that hepatic inflammation and fibrosis can occur independently of insulin resistance and hepatic steatosis. On the other hand, most of the well-characterized therapeutic means against MASLD/MASH implicate induction of hepatic CEACAM1 expression. For instance, caloric restriction ameliorates MASH phenotype and hepatic fibrosis in rats with low aerobic capacity, partly by inducing their hepatic CEACAM1 expression^[86]^. PPARγ and GLP-1 receptor agonists induce hepatic *Ceacam1* transcription by their direct binding to the well-conserved PPRE-RXR consensus sequence on its promoter^[83]^. This raises the possibility that inducing CEACAM1 could be a valid therapeutic target. Further studies are needed to explore whether hepatic fibrosis could similarly stem from deleting *Ceacam1* in other liver cells such as immune cells. This would ascertain a common underlying mechanism of CEACAM1’s prevention of hepatic fibrosis marked by limited off-target effects.

## References

[1] Byrne CD, Targher G. MASLD, MAFLD, or NAFLD criteria: have we re-created the confusion and acrimony surrounding metabolic syndrome? Metab Target Organ Damage 2024;4:10.

[2] Younossi ZM. Non-alcoholic fatty liver disease-a global public health perspective. J Hepatol 2019;70:531–44. 30414863 10.1016/j.jhep.2018.10.033

[3] Wang S, Friedman SL. Found in translation-fibrosis in metabolic dysfunction-associated steatohepatitis (MASH). Sci Transl Med 2023;15:eadi0759. 10.1126/scitranslmed.adi0759 PMC10671253 37792957

[4] Goldner D, Lavine JE. Nonalcoholic fatty liver disease in children: unique considerations and challenges. Gastroenterology 2020;158:1967–83.e1. 32201176 10.1053/j.gastro.2020.01.048

[5] Díaz LA, Villota-Rivas M, Barrera F, Lazarus JV, Arrese M. The burden of liver disease in Latin America. Ann Hepatol 2024;29:101175. 37922988 10.1016/j.aohep.2023.101175

[6] Shaheen M, Pan D, Schrode KM, Reassessment of the hispanic disparity: hepatic steatosis is more prevalent in mexican americans than other hispanics. Hepatol Commun 2021;5:2068–79. 34558824 10.1002/hep4.1775 PMC8631095

[7] Shaheen M, Schrode KM, Pan D, Sex-specific differences in the association between race/ethnicity and NAFLD among US population. Front Med (Lausanne) 2021;8:795421. 34926533 10.3389/fmed.2021.795421 PMC8674562

[8] Driessen S, Francque SM, Anker SD, Metabolic dysfunction-associated steatotic liver disease and the heart. Hepatology 2023;Online ahead of print. 10.1097/HEP.0000000000000735 PMC12266800 38147315

[9] Yan L, Hu X, Wu S, Cui C, Zhao S. Association between the cardiometabolic index and NAFLD and fibrosis. Sci Rep 2024;14:13194. 38851771 10.1038/s41598-024-64034-3 PMC11162484

[10] Hirata A, Harada S, Iida M, Association of nonalcoholic fatty liver disease with arterial stiffness and its metabolomic profiling in japanese community-dwellers. J Atheroscler Thromb 2024;31:1031–47. 38311416 10.5551/jat.64616 PMC11224684

[11] Xu L, Hui AY, Albanis E, Human hepatic stellate cell lines, LX-1 and LX-2: new tools for analysis of hepatic fibrosis. Gut 2005;54:142–51. 15591520 10.1136/gut.2004.042127 PMC1774377

[12] Harrison SA, Bedossa P, Guy CD, ; MAESTRO-NASH Investigators. A phase 3, randomized, controlled trial of resmetirom in NASH with liver fibrosis. N Engl J Med 2024;390:497–509. 38324483 10.1056/NEJMoa2309000

[13] Sevastianova K, Kotronen A, Gastaldelli A, Genetic variation in PNPLA3 (adiponutrin) confers sensitivity to weight loss-induced decrease in liver fat in humans. Am J Clin Nutr 2011;94:104–11. 21525193 10.3945/ajcn.111.012369

[14] Wang CW, Lin HY, Shin SJ, The PNPLA3 I148M polymorphism is associated with insulin resistance and nonalcoholic fatty liver disease in a normoglycaemic population. Liver Int 2011;31:1326–31. 21745282 10.1111/j.1478-3231.2011.02526.x

[15] European Association for the Study of the Liver (EASL); European Association for the Study of Diabetes (EASD); European Association for the Study of Obesity (EASO). EASL-EASD-EASO clinical practice guidelines on the management of metabolic dysfunction-associated steatotic liver disease (MASLD). Obes Facts 2024;17:374–444. 38852583 10.1159/000539371 PMC11299976

[16] Newsome PN, Ambery P. Incretins (GLP-1 receptor agonists and dual/triple agonists) and the liver. J Hepatol 2023;79:1557–65. 37562748 10.1016/j.jhep.2023.07.033

[17] Puengel T, Tacke F. Pharmacotherapeutic options for metabolic dysfunction-associated steatotic liver disease: where are we today? Expert Opin Pharmacother 2024;25:1249–63. 38954663 10.1080/14656566.2024.2374463

[18] Armstrong MJ, Gaunt P, Aithal GP, Liraglutide safety and efficacy in patients with non-alcoholic steatohepatitis (LEAN): a multicentre, double-blind, randomised, placebo-controlled phase 2 study. Lancet 2016;387:679–90. 26608256 10.1016/S0140-6736(15)00803-X

[19] Loomba R, Abdelmalek MF, Armstrong MJ, ; NN9931-4492 investigators. Semaglutide 2·4 mg once weekly in patients with non-alcoholic steatohepatitis-related cirrhosis: a randomised, placebo-controlled phase 2 trial. Lancet Gastroenterol Hepatol 2023;8:511–22. 36934740 10.1016/S2468-1253(23)00068-7 PMC10792518

[20] Speliotes EK, Butler JL, Palmer CD, Voight BF, ; Hirschhorn JN; GIANT Consortium; MIGen Consortium; NASH CRN. PNPLA3 variants specifically confer increased risk for histologic nonalcoholic fatty liver disease but not metabolic disease. Hepatology 2010;52:904–12. 20648472 10.1002/hep.23768 PMC3070300

[21] Sookoian S, Pirola CJ. Meta-analysis of the influence of I148M variant of patatin-like phospholipase domain containing 3 gene (PNPLA3) on the susceptibility and histological severity of nonalcoholic fatty liver disease. Hepatology 2011;53:1883–94. 21381068 10.1002/hep.24283

[22] Kantartzis K, Peter A, Machicao F, Dissociation between fatty liver and insulin resistance in humans carrying a variant of the patatin-like phospholipase 3 gene. Diabetes 2009;58:2616–23. 19651814 10.2337/db09-0279 PMC2768178

[23] Petit JM, Guiu B, Masson D, Specifically PNPLA3-mediated accumulation of liver fat in obese patients with type 2 diabetes. J Clin Endocrinol Metab 2010;95:E430–6. 20826584 10.1210/jc.2010-0814

[24] Chen LZ, Xin YN, Geng N, Jiang M, Zhang DD, Xuan SY. PNPLA3 I148M variant in nonalcoholic fatty liver disease: demographic and ethnic characteristics and the role of the variant in nonalcoholic fatty liver fibrosis. World J Gastroenterol 2015;21:794–802. 25624712 10.3748/wjg.v21.i3.794 PMC4299331

[25] Lee CC, Haffner SM, Wagenknecht LE, Insulin clearance and the incidence of type 2 diabetes in Hispanics and African Americans: the IRAS family study. Diabetes Care 2013;36:901–7. 23223351 10.2337/dc12-1316 PMC3609510

[26] Shah MH, Piaggi P, Looker HC, Paddock E, Krakoff J, Chang DC. Lower insulin clearance is associated with increased risk of type 2 diabetes in native Americans. Diabetologia 2021;64:914–22. 33404681 10.1007/s00125-020-05348-5

[27] Matveyenko AV, Liuwantara D, Gurlo T, Pulsatile portal vein insulin delivery enhances hepatic insulin action and signaling. Diabetes 2012;61:2269–79. 22688333 10.2337/db11-1462 PMC3425431

[28] Lee WH, Najjar SM, Kahn CR, Hinds TD Jr. Hepatic insulin receptor: new views on the mechanisms of liver disease. Metabolism 2023;145:155607. 37271372 10.1016/j.metabol.2023.155607 PMC10330768

[29] Haeusler RA, McGraw TE, Accili D. Biochemical and cellular properties of insulin receptor signalling. Nat Rev Mol Cell Biol 2018;19:31–44. 28974775 10.1038/nrm.2017.89 PMC5894887

[30] Saltiel AR. Insulin signaling in health and disease. J Clin Invest 2021;131:142241. 33393497 10.1172/JCI142241 PMC7773347

[31] Najjar SM, Caprio S, Gastaldelli A. Insulin clearance in health and disease. Annu Rev Physiol 2023;85:363–81. 36260807 10.1146/annurev-physiol-031622-043133

[32] Meijer RI, Barrett EJ. The insulin receptor mediates insulin’s early plasma clearance by liver, muscle, and kidney. Biomedicines 2021;9:37. 33466380 10.3390/biomedicines9010037 PMC7824884

[33] Najjar SM, Perdomo G. Hepatic insulin clearance: mechanism and physiology. Physiology (Bethesda) 2019;34:198–215. 30968756 10.1152/physiol.00048.2018 PMC6734066

[34] Bergman RN, Kabir M, Ader M. The physiology of insulin clearance. Int J Mol Sci 2022;23:1826. 35163746 10.3390/ijms23031826 PMC8836929

[35] Bergman RN, Piccinini F, Kabir M, Kolka CM, Ader M. Hypothesis: role of reduced hepatic insulin clearance in the pathogenesis of type 2 diabetes. Diabetes 2019;68:1709–16. 31431441 10.2337/db19-0098 PMC6702636

[36] Tokarz VL, MacDonald PE, Klip A. The cell biology of systemic insulin function. J Cell Biol 2018;217:2273–89. 29622564 10.1083/jcb.201802095 PMC6028526

[37] Kolka CM, Bergman RN. The barrier within: endothelial transport of hormones. Physiology (Bethesda) 2012;27:237–47. 22875454 10.1152/physiol.00012.2012 PMC4423824

[38] Najjar SM, Philippe N, Suzuki Y, Insulin-stimulated phosphorylation of recombinant pp120/HA4, an endogenous substrate of the insulin receptor tyrosine kinase. Biochemistry 1995;34:9341–9. 7626603 10.1021/bi00029a009

[39] Najjar SM, Accili D, Philippe N, Jernberg J, Margolis R, Taylor SI. pp120/ecto-ATPase, an endogenous substrate of the insulin receptor tyrosine kinase, is expressed as two variably spliced isoforms. J Biol Chem 1993;268:1201–6. 8380406

[40] Sundberg U, Beauchemin N, Obrink B. The cytoplasmic domain of CEACAM1-L controls its lateral localization and the organization of desmosomes in polarized epithelial cells. J Cell Sci 2004;117:1091–104. 14970258 10.1242/jcs.00944

[41] Formisano P, Najjar SM, Gross CN, Receptor-mediated internalization of insulin. Potential role of pp120/HA4, a substrate of the insulin receptor kinase. J Biol Chem 1995;270:24073–7. 7592607 10.1074/jbc.270.41.24073

[42] Najjar SM, Yang Y, Fernström MA, Insulin acutely decreases hepatic fatty acid synthase activity. Cell Metab 2005;2:43–53. 16054098 10.1016/j.cmet.2005.06.001

[43] Najjar SM, Abdolahipour R, Ghadieh HE, Regulation of insulin clearance by non-esterified fatty acids. Biomedicines 2022;10:1899. 36009446 10.3390/biomedicines10081899 PMC9405499

[44] Osborne TF. Sterol regulatory element-binding proteins (SREBPs): key regulators of nutritional homeostasis and insulin action. J Biol Chem 2000;275:32379–82. 10934219 10.1074/jbc.R000017200

[45] Ter Horst KW, Vatner DF, Zhang D, Hepatic insulin resistance is not pathway selective in humans with nonalcoholic fatty liver disease. Diabetes Care 2021;44:489–98. 33293347 10.2337/dc20-1644 PMC7818337

[46] Zaharia OP, Antoniou S, Bobrov P, ; GDS Group. Reduced insulin clearance differently relates to increased liver lipid content and worse glycemic control in recent-onset type 2 and type 1 diabetes. Diabetes Care 2023;46:2232–9. 37874983 10.2337/dc23-1267 PMC10698223

[47] Ferrannini E The stunned beta cell: a brief history. Cell Metab 2010;11:349–52. 20444416 10.1016/j.cmet.2010.04.009

[48] Bojsen-Møller KN, Lundsgaard AM, Madsbad S, Kiens B, Holst JJ. Hepatic insulin clearance in regulation of systemic insulin concentrations-role of carbohydrate and energy availability. Diabetes 2018;67:2129–36. 30348819 10.2337/db18-0539

[49] Corkey BE. Banting lecture 2011: hyperinsulinemia: cause or consequence? Diabetes 2012;61:4–13. 22187369 10.2337/db11-1483 PMC3237642

[50] Pories WJ, Dohm GL. Diabetes: have we got it all wrong? Diabetes Care 2012;35:2438–42. 23173133 10.2337/dc12-0684 PMC3507594

[51] Tricò D, Galderisi A, Mari A, Intrahepatic fat, irrespective of ethnicity, is associated with reduced endogenous insulin clearance and hepatic insulin resistance in obese youths: a cross-sectional and longitudinal study from the Yale Pediatric NAFLD cohort. Diabetes Obes Metab 2020;22:1628–38. 32363679 10.1111/dom.14076 PMC8174801

[52] Pivovarova O, Bernigau W, Bobbert T, Hepatic insulin clearance is closely related to metabolic syndrome components. Diabetes Care 2013;36:3779–85. 24026549 10.2337/dc12-1203 PMC3816867

[53] Smith K, Taylor GS, Peeters W, Elevations in plasma glucagon are associated with reduced insulin clearance after ingestion of a mixed-macronutrient meal in people with and without type 2 diabetes. Diabetologia 2024;Online ahead of print. 10.1007/s00125-024-06249-7 PMC11519192 39138690

[54] Ghadieh HE, Russo L, Muturi HT, Hyperinsulinemia drives hepatic insulin resistance in male mice with liver-specific Ceacam1 deletion independently of lipolysis. Metabolism 2019;93:33–43. 30664851 10.1016/j.metabol.2019.01.008 PMC6401268

[55] Poy MN, Yang Y, Rezaei K, CEACAM1 regulates insulin clearance in liver. Nat Genet 2002;30:270–6. 11850617 10.1038/ng840

[56] Russo L, Muturi HT, Ghadieh HE, Liver-specific reconstitution of CEACAM1 reverses the metabolic abnormalities caused by its global deletion in male mice. Diabetologia 2017;60:2463–74. 28913658 10.1007/s00125-017-4432-y PMC5788286

[57] Watada H, Tamura Y. Impaired insulin clearance as a cause rather than a consequence of insulin resistance. J Diabetes Investig 2017;8:723–5. 10.1111/jdi.12717 PMC5668474 28752566

[58] Bril F, Lomonaco R, Orsak B, Relationship between disease severity, hyperinsulinemia, and impaired insulin clearance in patients with nonalcoholic steatohepatitis. Hepatology 2014;59:2178–87. 24777953 10.1002/hep.26988

[59] Ramakrishnan SK, Khuder SS, Al-Share QY, PPARα (peroxisome proliferator-activated receptor α) activation reduces hepatic CEACAM1 protein expression to regulate fatty acid oxidation during fasting-refeeding transition. J Biol Chem 2016;291:8121–9. 26846848 10.1074/jbc.M116.714014 PMC4825014

[60] Al-Share QY, DeAngelis AM, Lester SG, Forced hepatic overexpression of CEACAM1 curtails diet-induced insulin resistance. Diabetes 2015;64:2780–90. 25972571 10.2337/db14-1772 PMC4512217

[61] Russo L, Ghadieh HE, Ghanem SS, Role for hepatic CEACAM1 in regulating fatty acid metabolism along the adipocyte-hepatocyte axis. J Lipid Res 2016;57:2163–75. 27777319 10.1194/jlr.M072066 PMC5321226

[62] Lee W The CEACAM1 expression is decreased in the liver of severely obese patients with or without diabetes. Diagn Pathol 2011;6:40. 21569294 10.1186/1746-1596-6-40 PMC3104481

[63] Zaidi S, Asalla S, Muturi HT, Loss of CEACAM1 in hepatocytes causes hepatic fibrosis. Eur J Clin Invest 2024;54:e14177. 38381498 10.1111/eci.14177 PMC11153018

[64] DeAngelis AM, Heinrich G, Dai T, Carcinoembryonic antigen-related cell adhesion molecule 1: a link between insulin and lipid metabolism. Diabetes 2008;57:2296–303. 18544705 10.2337/db08-0379 PMC2518480

[65] Ghosh S, Kaw M, Patel PR, Mice with null mutation of Ceacam I develop nonalcoholic steatohepatitis. Hepat Med 2010;2010:69–78. 21949477 10.2147/HMER.S8902 PMC3177946

[66] Helal RA, Russo L, Ghadieh HE, Regulation of hepatic fibrosis by carcinoembryonic antigen-related cell adhesion molecule 1. Metabolism 2021;121:154801. 34058224 10.1016/j.metabol.2021.154801 PMC8286970

[67] Xu E, Dubois MJ, Leung N, Targeted disruption of carcinoembryonic antigen-related cell adhesion molecule 1 promotes diet-induced hepatic steatosis and insulin resistance. Endocrinology 2009;150:3503–12. 19406938 10.1210/en.2008-1439

[68] Brunt EM, Kleiner DE, Carpenter DH, ; American Association for the Study of Liver Diseases NASH TASK FORce. NAFLD: reporting histologic findings in clinical practice. Hepatology 2021;73:2028–38. 33111374 10.1002/hep.31599

[69] Friedman SL. Mechanisms of hepatic fibrogenesis. Gastroenterology 2008;134:1655–69. 18471545 10.1053/j.gastro.2008.03.003 PMC2888539

[70] Jophlin LL, Koutalos Y, Chen C, Shah V, Rockey DC. Hepatic stellate cells retain retinoid-laden lipid droplets after cellular transdifferentiation into activated myofibroblasts. Am J Physiol Gastrointest Liver Physiol 2018;315:G713–21. 30024770 10.1152/ajpgi.00251.2017 PMC6293250

[71] Hellemans K, Michalik L, Dittie A, Peroxisome proliferator-activated receptor-beta signaling contributes to enhanced proliferation of hepatic stellate cells. Gastroenterology 2003;124:184–201. 12512042 10.1053/gast.2003.50015

[72] Kostadinova R, Montagner A, Gouranton E, GW501516-activated PPARβ/δ promotes liver fibrosis via p38-JNK MAPK-induced hepatic stellate cell proliferation. Cell Biosci 2012;2:34. 23046570 10.1186/2045-3701-2-34 PMC3519722

[73] Berry DC, Noy N. All-trans-retinoic acid represses obesity and insulin resistance by activating both peroxisome proliferation-activated receptor beta/delta and retinoic acid receptor. Mol Cell Biol 2009;29:3286–96. 19364826 10.1128/MCB.01742-08 PMC2698724

[74] Forman BM, Chen J, Evans RM. Hypolipidemic drugs, polyunsaturated fatty acids, and eicosanoids are ligands for peroxisome proliferator-activated receptors alpha and delta. Proc Natl Acad Sci U S A 1997;94:4312–7. 9113986 10.1073/pnas.94.9.4312 PMC20719

[75] Muturi HT, Ghadieh HE, Asalla S, Conditional deletion of CEACAM1 in hepatic stellate cells causes their activation. Mol Metab 2024;88:102010. 39168268 10.1016/j.molmet.2024.102010 PMC11403062

[76] Muturi HT, Ghadieh HE, Abdolahipour R, Loss of CEACAM1 in endothelial cells causes hepatic fibrosis. Metabolism 2023;144:155562. 37088122 10.1016/j.metabol.2023.155562 PMC10330196

[77] Wang Y, van Boxel-Dezaire AH, Cheon H, Yang J, Stark GR. STAT3 activation in response to IL-6 is prolonged by the binding of IL-6 receptor to EGF receptor. Proc Natl Acad Sci U S A 2013;110:16975–80. 24082147 10.1073/pnas.1315862110 PMC3801081

[78] Najjar SM, Russo L. CEACAM1 loss links inflammation to insulin resistance in obesity and non-alcoholic steatohepatitis (NASH). Semin Immunopathol 2014;36:55–71. 24258517 10.1007/s00281-013-0407-3 PMC3946532

[79] Abou-Rjaily GA, Lee SJ, May D, CEACAM1 modulates epidermal growth factor receptor-mediated cell proliferation. J Clin Invest 2004;114:944–52. 15467833 10.1172/JCI21786 PMC518664

[80] Lee SJ, Heinrich G, Fedorova L, Development of nonalcoholic steatohepatitis in insulin-resistant liver-specific S503A carcinoembryonic antigen-related cell adhesion molecule 1 mutant mice. Gastroenterology 2008;135:2084–95. 18848945 10.1053/j.gastro.2008.08.007 PMC2784638

[81] Lester SG, Russo L, Ghanem SS, Hepatic CEACAM1 over-expression protects against diet-induced fibrosis and inflammation in white adipose tissue. Front Endocrinol (Lausanne) 2015;6:116. 26284027 10.3389/fendo.2015.00116 PMC4522571

[82] Muturi HT, Khuder SS, Ghadieh HE, Insulin sensitivity is retained in mice with endothelial loss of carcinoembryonic antigen cell adhesion molecule 1. Cells 2021;10:2093. 34440862 10.3390/cells10082093 PMC8394790

[83] Ghadieh HE, Muturi HT, Russo L, Exenatide induces carcinoembryonic antigen-related cell adhesion molecule 1 expression to prevent hepatic steatosis. Hepatol Commun 2018;2:35–47. 29404511 10.1002/hep4.1117 PMC5776867

[84] Horst AK, Najjar SM, Wagener C, Tiegs G. CEACAM1 in liver injury, metabolic and immune regulation. Int J Mol Sci 2018;19:3110. 30314283 10.3390/ijms19103110 PMC6213298

[85] Nakamura K, Kageyama S, Kaldas FM, Hepatic CEACAM1 expression indicates donor liver quality and prevents early transplantation injury. J Clin Invest 2020;130:2689–704. 32027621 10.1172/JCI133142 PMC7190917

[86] Bowman TA, Ramakrishnan SK, Kaw M, Caloric restriction reverses hepatic insulin resistance and steatosis in rats with low aerobic capacity. Endocrinology 2010;151:5157–64. 20861239 10.1210/en.2010-0176 PMC2954714

